# Milk Composition Is Predictive of Low Milk Supply Using Machine Learning Approaches †

**DOI:** 10.3390/diagnostics15020191

**Published:** 2025-01-15

**Authors:** Xuehua Jin, Ching Tat Lai, Sharon L. Perrella, Xiaojie Zhou, Ghulam Mubashar Hassan, Jacki L. McEachran, Zoya Gridneva, Nicolas L. Taylor, Mary E. Wlodek, Donna T. Geddes

**Affiliations:** 1 School of Molecular Sciences, The University of Western Australia, Crawley, WA 6009, Australia; xuehua.jin@research.uwa.edu.au (X.J.); ching-tat.lai@uwa.edu.au (C.T.L.); sharon.perrella@uwa.edu.au (S.L.P.); xiaojie.zhou@uwa.edu.au (X.Z.); jacki.mceachran@uwa.edu.au (J.L.M.); zoya.gridneva@uwa.edu.au (Z.G.); nicolas.taylor@uwa.edu.au (N.L.T.); m.wlodek@unimelb.edu.au (M.E.W.); 2 UWA Centre for Human Lactation Research and Translation, Crawley, WA 6009, Australia; 3 ABREAST Network, Perth, WA 6000, Australia; 4 School of Physics, Mathematics and Computing, The University of Western Australia, Crawley, WA 6009, Australia; ghulam.hassan@uwa.edu.au; 5 ARC Training Centre for Next-Gen Technologies in Biomedical Analysis, The University of Western Australia, Crawley, WA 6009, Australia; 6 Department of Obstetrics, Gynaecology and Newborn Health, The University of Melbourne, Melbourne, VIC 3010, Australia

**Keywords:** human milk, lactation, breastfeeding, biomarkers, milk composition, milk supply, predictive model, machine learning

## Abstract

**Background/Objectives:** The causes of low milk supply are multifactorial, including factors such as gene mutations, endocrine disorders, and infrequent milk removal. These factors affect the functional capacity of the mammary gland and, potentially, the concentrations of milk components. This study aimed to investigate the differences in milk composition between mothers with low and normal milk supply and develop predictive machine learning models for identifying low milk supply. **Methods:** Twenty-four-hour milk production measurements were conducted using the test-weigh method. An array of milk components was measured in 58 women with low milk supply (<600 mL/24 h) and 106 with normal milk supply (≥600 mL/24 h). Machine learning algorithms were employed to develop prediction models integrating milk composition and maternal and infant characteristics. **Results:** Among the six machine learning algorithms tested, deep learning and gradient boosting machines methods had the best performance metrics. The best-performing model, incorporating 14 milk components and maternal and infant characteristics, achieved an accuracy of 87.9%, an area under the precision-recall curve (AUPRC) of 0.893, and an area under the receiver operating characteristic curve (AUC) of 0.917. Additionally, a simplified model, optimised for clinical applicability, maintained a reasonable accuracy of 78.8%, an AUPRC of 0.776, and an AUC of 0.794. **Conclusions:** These findings demonstrate the potential of machine learning models to predict low milk supply with high accuracy. Integrating milk composition and maternal and infant characteristics offers a practical approach to identify women at risk of low milk supply, facilitating timely interventions to support breastfeeding and ensure adequate infant nutrition.

## 1. Introduction

Evidence supporting human milk as the optimal personalised nutrition for infants is unequivocal. Indeed, global organisations such as the World Health Organisation (WHO) and the United Nations International Children’s Emergency Fund (UNICEF) recommend exclusive breastfeeding for the first six months of life and continued breastfeeding for two years and beyond [1]. Despite strong support for breastfeeding, many women fail to meet the recommendations, with perceived low milk supply (LMS) and pain during breastfeeding ranking as the primary reasons for stopping breastfeeding earlier than planned [2,3,4]. Whilst there is the long-held belief that the majority of women can produce sufficient breast milk to meet their infants’ nutritional needs, recent estimates of LMS are as high as 10–15% but maybe even higher given the increasing prevalence of metabolic health conditions that may impact milk production (MP) [5,6]. In Australia, breastfeeding initiation rates are high (>90%) [7]. However, exclusive breastfeeding rates drop sharply in the subsequent months. By one month of age, only 60% of infants were exclusively breastfed in 2010, with a similar rate of 59% in 2021 [7,8]. This means approximately 40% of infants at one month received formula or other milk supplementation, highlighting ongoing challenges despite public health efforts to support breastfeeding.

Factors influencing MP include hormonal drivers essential to promoting organised breast development in pregnancy and the initiation and maintenance of MP during lactation [9]. Causes of LMS include anatomical factors such as breast surgery [10] and nipple piercing [11], genetic anomalies such as mutations in zinc transporters [12,13], and mammary hypoplasia due to deficiencies or dysregulation of oestrogen and progesterone receptors [14,15]. Inadequate milk removal, such as poor management of breastfeeding and mother-infant separation, can also contribute to LMS [16]. Additionally, metabolic health disorders, such as maternal obesity, diabetes, and polycystic ovary syndrome (PCOS), can interfere with the endocrine system and impact breast development and lactation [17]. It is noteworthy that maternal factors affecting MP can also influence milk composition. For example, higher concentrations of fat in mature milk has been reported in women with overweight or obesity [18]. Women with type 2 diabetes or gestational diabetes mellitus (GDM) have been shown to have a slower increase in human milk citrate concentrations [19,20], indicating delayed secretory activation. Moreover, a murine model has illustrated that the absence of the zinc transporter ZnT2 results in decreased milk protein, fat, lactose, and zinc compared to their wild-type counterparts [21]. Given these considerations, we hypothesised that specific milk components may serve as biomarkers indicative of LMS and provide an opportunity to predict LMS in time to ensure adequate nutritional intake for infants.

Many studies have shown that delayed secretory activation is a risk factor for poor lactation outcomes [22,23]. After birth, the rapid withdrawal of progesterone coincides with an increase in milk synthesis. This process occurs within 48–72 h and is accompanied by marked changes in milk component concentrations, such as a decrease in protein and sodium (Na) and an increase in lactose and citrate [22]. These changes result from the closure of tight junctions between the mammary epithelial cells, limiting the passage of components between the maternal circulation and the alveoli [24]. Efforts have been made to use these secretory activation biomarkers to predict subsequent lactation. Studies involving milk collected during the first 14 postpartum days showed that an elevated sodium-to-potassium (Na:K) ratio was predictive of perceived insufficient milk supply and early weaning [25,26]. Hoban et al. [27] reported a correlation between milk volume produced by pump-dependent mothers of preterm infants and milk Na concentration and milk Na:K ratio. However, limited studies have measured 24-h MP or analysed milk samples beyond two weeks postpartum, leaving uncertainty about whether milk components during established lactation can serve as biomarkers for LMS. Additionally, questions remain regarding which phenotype poses the highest risk for LMS, presenting a challenge in implementing targeted tests for clinical diagnosis and support.

Conventional statistical methods have been widely used to analyse biomarkers associated with lactation outcomes, primarily focusing on linear relationships between variables. While these methods have provided valuable insights, they are often limited in capturing complex, non-linear interactions and may overlook nuanced patterns in the data [28]. Machine learning offers a complementary approach, excelling in prediction accuracy and discovering intricate associations [28]. With advancements in model interpretability tools, machine learning approaches enable feature importance ranking and relationship estimation between predictors and outcomes, enhancing interpretability and utility in clinical and research settings [29]. Several studies have explored machine learning models to predict feeding mode and breastfeeding behaviours [30,31,32,33], but to the best of our knowledge, we present the first study using various machine learning algorithms to explore the possibility that milk composition is predictive of LMS.

In this study, we conducted 24-h MP measurements and investigated various milk component concentrations. Importantly, we applied machine learning to establish predictive models for identifying LMS based on milk composition and maternal and infant characteristics. These findings provide an evidence base for identifying women at risk of LMS, allowing timely intervention and management to support breastfeeding and ensure the adequate nutritional intake of infants.

## 2. Materials and Methods

### 2.1. Participants

This study involved 164 English-speaking breastfeeding mothers of full-term infants aged 1–6 months. All mothers were recruited from the Perth metropolitan area, Western Australia, between 2017 and 2023, including women with no MP concerns and women with potential LMS. Recruitment was conducted via social media posts on Instagram and Facebook pages targeting women of childbearing age. The study was conducted in accordance with the Declaration of Helsinki and was approved by The University of Western Australia (UWA) Human Research Ethics Committee (2019/RA/4/20/6134). Informed consent was obtained from all participants.

### 2.2. Data Collection

Given the stability in daily infant intake of human milk between 1–6 months postpartum [34], MP measurements were conducted within this temporal range. Mothers test-weighed their infants in their own home, before and after each breastfeeding from each breast on an electronic Baby Weigh Scale (±2.0 g; Electronic Baby Weigh Scale, Medela Inc., McHenry, IL, USA) for all feeds in a 24 ± 4-h period [35]. The weights of milk collection bottles before and after expressions from each breast were also included when mothers chose to pump. Milk removal frequency was calculated as the sum of breastfeeding and milk expression times over 24 h, when applicable. MP expressed in grams was considered equivalent to millilitres as the density of milk is 1.03 g/mL [36]. 24-h MP was calculated with the formula below [37], where *v_i_* is the volume of each breastfeed/expression, *N* is the total number of breastfeeds and/or expressions, and *T* is the elapsed time from the end of the first feed until the end of the last feed.

$$MP=\sum_{i=2}^{N}v_{i}\frac{24}{T}$$

LMS was defined as less than 600 mL, determined through the clinical use of test weighing of infant milk intake, which indicated that the typical 24-h milk intake of fully breastfed infants was 788 ± 160 mL, with few taking <600 mL [35].

Mothers completed questionnaires providing demographic and anthropometric details, including maternal age, height, pre-pregnancy and postpartum bra size, parity, delivery mode, infant birth weight, and infant sex. Maternal current weight was self-reported through the background questionnaires, while infant current weight was measured at home using Baby Weigh Scales with an accuracy of ±2.0 g (Medela Inc., McHenry, IL, USA). Maternal BMI was calculated as kg/m^2^, and maternal breast volume (BV) of one breast was calculated based on bra cup and band size, as previously described [37]. Maternal BV growth during pregnancy was calculated as postpartum BV minus pre-pregnancy BV. Infant weight for age z-score (WAZ) was calculated using the R (version 4.2.2) package ‘Anthro’ according to the World Health Organization recommendations [38].

### 2.3. Sample Collection and Analysis

A small milk sample (around 5 mL) was collected into 5-mL polypropylene plastic vials (Disposable Products, Adelaide, Australia) within 1 week of MP measurements. Samples were stored at 4 °C for a maximum of 24 h before being transported on ice to the lab. Upon arrival, the samples were aliquoted at 1 mL each and stored at −80 °C until analysis.

***Fat analysis:*** Whole milk samples were thawed at room temperature, transferred into glass capillaries, sealed, and centrifuged at 12,000× *g* for 10 min. After centrifugation, the cream content of the milk samples was measured using the creamatocrit method. The fat concentration of each sample (g/L), was calculated as 5.917 × creamatocrit + 3.968 [39]. Glass capillaries were then cut to remove fat content. The remaining skim milk was collected into new labelled sample tubes and stored at −20 °C.

Frozen skim milk samples were thawed at room temperature to analyse lactose, protein, and citrate concentrations. All standards, samples, and qualitative control samples were processed on 96-well plates and analysed in duplicate.

***Lactose analysis:*** The lactose concentration in skim milk was measured using the Megazyme lactose kit (K-LOLAC, Megazyme, Wicklow, Ireland). This assay recovered 100.56 ± 6.58% in 8 sets, and CV was 6.55%. The detection limit of this assay was 5.824 g/L.

***Protein analysis:*** Total protein concentrations in skim milk were measured using the Bio-Rad DC Protein Assay Kit (Bio-Rad Laboratories, Hercules, CA, USA), adapted from the Lowry method [40], with human milk protein standard prepared according to the Kjeldahl method [41]. The recovery of a known amount of protein added to the milk samples was 98.0 ± 2.0% (*n* = 8), with a detection limit of 0.079 g/L. Casein and whey protein were separated by the method described by Kunz et al. [42] and Khan et al. [43], followed by a Lowry protein assay to measure their concentrations.

***Citrate analysis:*** Citrate concentration was determined using deproteinised skim milk and an enzymatic spectrophotometric assay, as previously described [44]. Recovery was 96.6 ± 2.7% in 8 sets, CV was 3.08%, and the detection limit of this assay was 0.06 mM. The citrate concentrations were expressed as mg/L, using the molecular weight of 192.1 g/mol to convert the results from mM.

***Mineral analysis:*** Eight elements were measured in mineral analysis: Na, K, calcium (Ca), copper (Cu), zinc (Zn), magnesium (Mg), phosphorus (P), and iron (Fe). Whole milk samples were thawed and digested with 65% nitric acid [45]. Milk powder from the National Institute of Standards and Technology (NIST) were used to control the analytical accuracy of the results obtained. Samples were analysed using inductively coupled plasma optical emission spectrometry (ICP-OES) with recoveries ranging from 97.36% to 102.2%, and CVs were 3.5% for Na, 4.8% for K, 17.4% for Mg, 2.3% for Zn, 3.5% for Ca, and 6.2% for Fe.

### 2.4. Statistical Analysis

***Descriptive statistics:*** Mean and standard deviation, as well as frequencies and percentages, were used to describe the distribution of continuous variables and categorical variables, respectively. To compare maternal and infant characteristics and milk composition between LMS and NMS groups, the Fisher exact test was used for categorical variables. For continuous variables, *t*-test was applied to normally distributed data, while the Mann–Whitney U test was employed for non-normally distributed data. The normality of continuous variables was assessed using the Shapiro–Wilk test. The significance level was set at *p* < 0.05, and all analyses were carried out in R Statistical Software 4.2.2 (R Foundation for Statistical Computing, Vienna, Austria).

**Figure 1:**
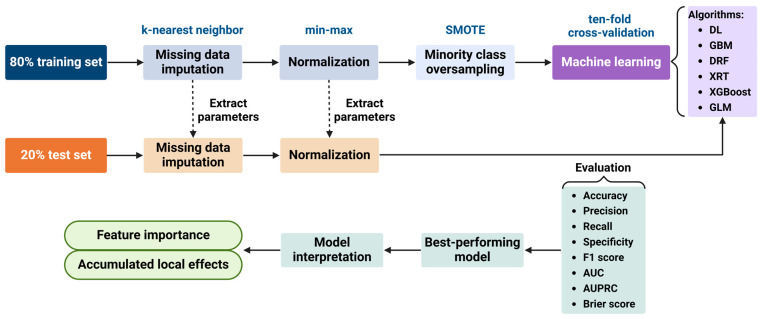
Schematic illustrating the development of predictive models using machine learning techniques. AUC: area under the receiver operating characteristic curve; AUPRC: area under the precision-recall curve; DL: deep learning; DRF: distributed random forest; GBM: gradient boosting machine; GLM: generalised linear model; SMOTE: synthetic minority oversampling technique; XGBoost: extreme gradient boosting; XRT: extremely randomised trees.

***Data pre-processing and model development:*** Pairwise correlations and variance inflation factor (VIF) were used to evaluate multicollinearity among predictors before the machine learning approach. Figure 1 shows the workflow of the predictive models development using machine learning techniques. The dataset was randomly split into a training set (80%) and a test set (20%). To avoid data leakage, missing value imputation and data normalisation were conducted in the training set first; then, the same parameters were applied to the test set. Missing values were imputed by the k-nearest neighbour using the ‘kNN’ function of R package ‘VIM’. Numeric variables were rescaled to the range [0, 1] by min–max normalisation, where x is the original value and x′ is the normalised value:

$$x\prime =\frac{x-min(x)}{max(x)-min(x)}$$

To prevent the model from overfitting to the majority class and ensure that models can identify patterns relevant to the minority class, we used the ‘synthetic minority oversampling technique (SMOTE)’ function from the R package ‘smotefamily’ to generate balanced training set by oversampling the minority class. We developed multiple machine learning models predicting LMS with three different sets of predictors: (A) milk components only, (B) milk components combined with maternal and infant characteristics, and (C) readily measurable milk components (fat, lactose, total protein, Na, and K) [46,47] combined with maternal and infant characteristics to maximise practical applicability. Automated machine learning (AutoML, ‘h2o.automl’ function in the R package ‘h2o’) was applied to identify the best-performing algorithm for predicting LMS. AutoML trained and cross-validated a variety of machine learning algorithms, including deep learning (DL), gradient boosting machine (GBM), distributed random forest (DRF), extremely randomised trees (XRT), extreme gradient boosting (XGBoost), and generalised linear model (GLM). We performed a 10-fold cross-validation method to train the AutoML models on the pre-processed training set and used the holdout test set to validate their performances. We used the area under the precision-recall curve (AUPRC) as the main metric to select the optimal algorithm based on the test set performance, which reflects how the model will generalise to new data.

***Model interpretation:*** We used the R package ‘iml’ to interpret the best-performing model for LMS. Feature importance was computed using the log-loss metric to assess the relative contribution of each predictor to the model’s performance. To further explore the relationship between key predictors and the likelihood of LMS, we generated accumulated local effect (ALE) plots for the top ten predictors. ALE plots estimate the average effect of a feature on model predictions by calculating changes in predictions when the feature is altered within small intervals while accounting for interactions and correlations with other predictors [29].

## 3. Results

### 3.1. Descriptive Statistics

A summary of maternal and infant characteristics and human milk composition is presented in Table 1. Fifty-eight (35.4%) participants were classified as LMS (<600 mL/24 h), while 106 (64.6%) participants were classified as NMS (≥600 mL/24 h). Women in the NMS group were significantly younger than those in the LMS group (*p* = 0.014). Infant WAZ (*p* = 0.004) and exclusive breastfeeding rate (*p* < 0.001) were significantly higher in the NMS group. Human milk fat (*p* = 0.020) and citrate (*p* < 0.001) concentrations were significantly higher in the NMS group, while lactose (*p* < 0.001) concentration was significantly higher in the LMS group.

### 3.2. Model Performance

**Figure 2:**
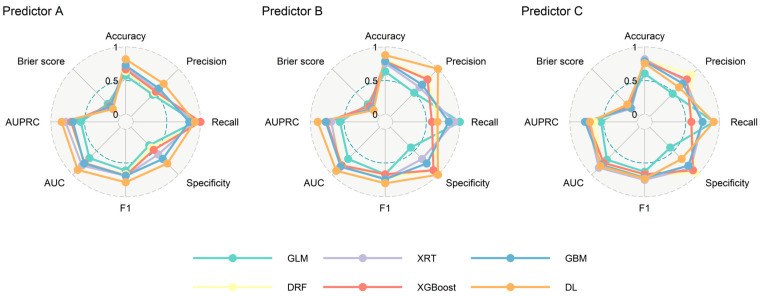
Comparisons of performances in test set for different predictors using different algorithms. AUC: area under the receiver operating characteristic curve; AUPRC: area under the precision-recall curve; DL: deep learning; DRF: distributed random forest; GBM: gradient boosting machine; GLM: generalised linear model; XGBoost: extreme gradient boosting; XRT: extremely randomised trees.

None of the pairwise correlation coefficients exceeded 0.8, and the calculated VIF for each predictor was lower than 5, suggesting the multicollinearity is not a significant concern in our dataset (Supplementary Materials Figure S1). Multiple machine learning models were developed to predict LMS, each employing three sets of predictors: Predictor A used all 14 milk components measured in the study; Predictor B incorporated maternal and infant characteristics alongside the variable in Predictor A, providing a broader dataset for prediction; and Predictor C prioritised applicability by using a reduced set of readily measurable prediction variables, including macronutrients, Na, K, and maternal and infant characteristics. Figure 2 compares the performance of various machine learning algorithms for each test set of Predictors. Detailed cross-validation results are provided in the Supplementary Materials Table S1. For Predictors A and B, DL consistently demonstrated the highest performance across multiple metrics, including accuracy, precision, specificity, F1-score, area under the receiver operating characteristic curve (AUC), and AUPRC, and it also had the lowest Brier score, indicating its superior overall performance. For Predictor C, several algorithms performed similarly, however, GBM was found to be the optimal algorithm based on its highest AUPRC value, which is particularly relevant given the imbalanced nature of the LMS outcome. Across all three set of predictors, DL, GBM, and XGBoost consistently ranked among the top-performing algorithms of the six evaluated. In contrast, GLM consistently showed the poorest performance for all three predictors. This underperformance highlights the limitations of GLM in capturing the non-linear and interactive effects of predictors, which are likely critical for predicting LMS.

**Figure 3:**
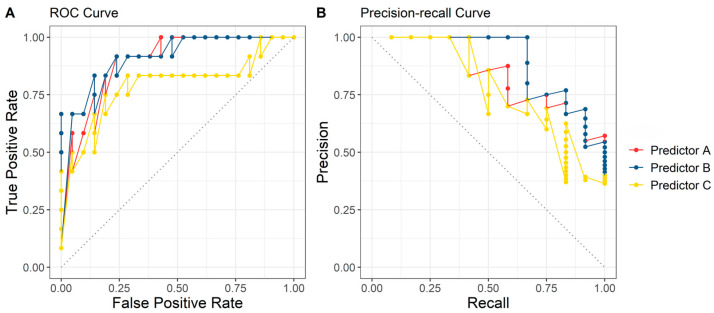
Receiver operating characteristic (ROC) curves and precision-recall (PR) curves for three set of predictors using their optimal respective machine learning algorithms. (**A**) ROC curve; (**B**) PR curve.

Figure 3 shows the receiver operating characteristic (ROC) curves and precision-recall (PR) curves for three sets of predictors using their respective optimal machine learning algorithms. In Figure 3A, Predictor B achieved the highest AUC (0.917), indicating excellent discrimination between LMS and NMS. In Figure 3B, Predictor B also demonstrated the highest AUPRC (0.893), reflecting its ability to maintain high precision across a wide range of recall values. These results highlight the robustness and reliability of Predictor B as the most balanced set of prediction variables. On the other hand, while Predictor C exhibited lower predictive performance than Predictors A and B, its reliance on fewer and more accessible prediction variable makes it highly applicable in clinical practice.

### 3.3. Prediction Variables Interpretation

**Figure 4:**
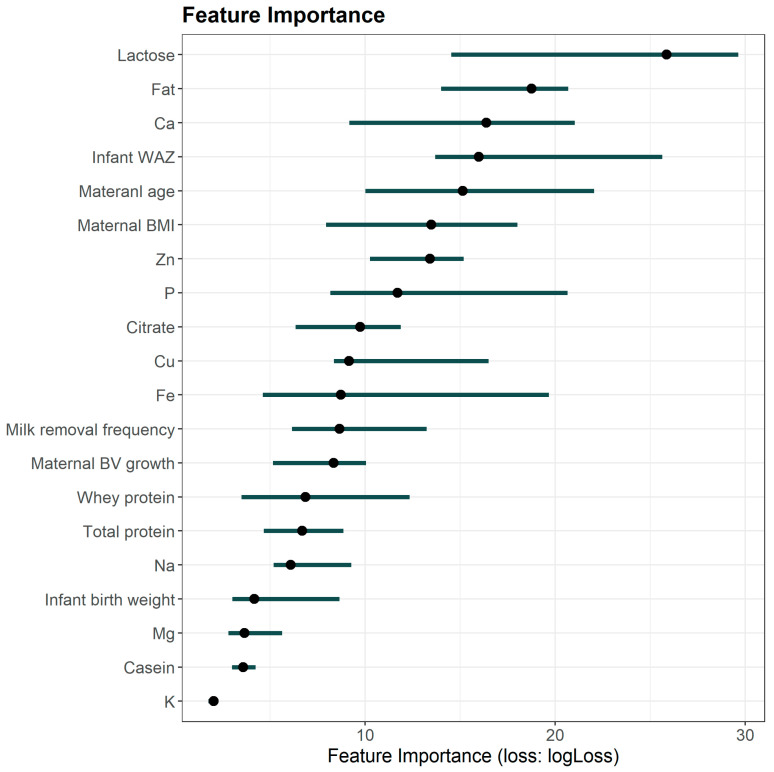
Feature importance derived from the best-performing model (Predictor B). BMI: body mass index; BV: breast volume; Ca: calcium; Cu: copper; Fe: iron; K: potassium; Mg: magnesium; Na: sodium; P: phosphorus; WAZ: weight-for-age z-score.

Feature importance was evaluated using the permutation approach, where the values of a given feature were randomly shuffled across the dataset while keeping other features unchanged. This process disrupts the relationship between the shuffled feature and the target variable, and the resulting increase in the prediction error quantifies the importance of that feature. Figure 4 presents the feature importance derived from Predictor B, which incorporated the greatest number of prediction variables and achieved the highest overall performance. Among milk composition variables, lactose, fat, and Ca emerged as the most influential prediction variables. In addition, infant WAZ, maternal age, and maternal BMI were identified as the most important maternal and infant characteristics.

**Figure 5:**
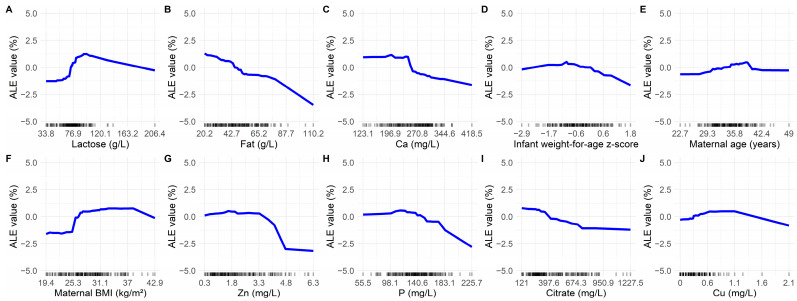
Feature effects on the probability of low milk supply. (**A**) Lactose; (**B**) Fat; (**C**) Ca; (**D**) Infant weight-for-age z-score; (**E**) Maternal age; (**F**) Maternal BMI; (**G**) Zn; (**H**) P; (**I**) Citrate; (**J**) Cu. ALE: accumulated local effect; BMI: body mass index; Ca: calcium; Cu: copper; P: phosphorus; Zn: zinc.

Figure 5 displays ALE plots for the top 10 prediction variables identified in Predictor B, illustrating their relationships with the likelihood of LMS. Among the milk components, lactose (Figure 5A) demonstrated a positive relationship with LMS likelihood up to approximately 100 g/L, where the effect began to decline at higher concentrations. Fat (Figure 5B) and citrate (Figure 5I) showed a nearly linear negative effect on LMS likelihood. Ca (Figure 5C), Zn (Figure 5G), and P (Figure 5H) exhibited a threshold effect, where their impact on LMS likelihood became pronounced at certain concentrations. Cu (Figure 5J) is mildly positively associated with LMS probability over the range of its densely distributed data. For maternal and infant characteristics, infant WAZ (Figure 5D) showed a negative association with the likelihood of LMS, with the most substantial effect observed at scores above 0. Similarly, maternal BMI (Figure 5F) demonstrated a threshold effect, with the probability of LMS sharply increasing at BMI levels exceeding 25 kg/m^2^.

## 4. Discussion

This study is the first to our knowledge to investigate milk composition during established lactation and explore its potential to predict LMS. Our findings suggest the feasibility of classifying LMS using compositional biomarkers obtained from a single collection, thus overcoming the usual challenges associated with time-consuming MP measurement protocols. Notably, integrating accessible maternal and infant characteristics into the prediction model based on milk composition enhances its predictive performance. Restricting prediction models to fewer biomarkers, anthropometrics, and demographics suggests a simplified protocol for future work and clinical application.

Our machine learning models achieved impressive accuracy and identified a broader range of potential biomarkers for LMS compared to previous studies [25,26,27]. In the best-performing machine learning model, lactose emerged as the most important predictor and was positively associated with the likelihood of LMS in the densely distributed data range. This finding seems unexpected at first glance, as lactose is the primary osmotic component of human milk, facilitating water transport into milk and influencing milk volume [48]. Previous studies have also reported higher lactose concentrations associated with increased MP [49,50]. However, when considering the total lactose production (calculated as lactose concentration × MP), the total lactose amount produced by mothers with LMS is significantly lower than that of mothers with NMS, even if lactose concentrations are higher in the LMS group. This observation supports the notion that higher lactose concentrations in LMS do not contradict prior findings. Lactose synthesis occurs in the mammary epithelial cells using glucose absorbed from the bloodstream [51]. Hence, differences in lactose between the two groups might reflect maternal metabolic conditions affecting plasma glucose availability and its uptake rate by the mammary gland [52]. While plasma glucose concentration is tightly regulated by insulin, studies in animal models suggest that mammary glucose uptake is independent of insulin and is instead stimulated by growth hormone and thyroxine [53,54,55]. These metabolic hormones are also essential for mammary gland development and lactation [56]. Unfortunately, plasma samples and complete maternal metabolic data were unavailable in this cohort to support this rationale.

Fat ranked as the second most robust biomarker contributing to prediction accuracy, with negative associations with the likelihood of LMS. Around 98% of the total fat concentration in human milk is triglycerides [57]. The mammary gland produces triglycerides from fatty acids derived from the diet, the body fat stores or is synthesised de novo in the smooth endoplasmic reticulum of alveolar cells [58]. Recent evidence showed that mothers with LMS at 2–10 weeks postpartum had significantly lower concentrations of long-chain fatty acids in milk, resulting from the insufficient transfer of fatty acids from circulation to the mammary gland [59]. Prolactin also plays a pivotal role in mammary fat production by stimulating key enzymes, such as lipoprotein lipase [60], pyruvate dehydrogenase [61], and fatty acid synthase [62]. Suckling is the primary physiological stimulus affecting prolactin secretion [63]. However, reduced prolactin response to suckling has been linked to lower lactogenesis and early lactation failure in women with obesity [64]. Therefore, such maternal endocrine abnormalities might contribute to the reduced milk fat concentration reported in mothers with LMS.

Citrate was also identified as a key predictor, showing negative associations with LMS likelihood. This aligns with prior studies indicating that human milk citrate exhibited a slower increase during the first week postpartum among women at higher risk of LMS [19,20]. Citrate is a key intermediate in the tricarboxylic acid (TCA) cycle, central to cellular energy production [65]. Hence, the higher citrate concentration observed in NMS suggests enhanced metabolic activity within the mammary gland, as the citrate present in milk originates from citrate formed within the mammary epithelial cells rather than from the bloodstream [66]. Citrate also acts as a chelator of calcium ions and plays a role in maintaining the appropriate ionic strength of milk, which is essential for casein micelle stability [67]. Moreover, milk citrate is related to de novo synthesis of fatty acids in the mammary gland [68,69], further linking it to the higher fat concentration observed in the milk of mothers with NMS.

The observed threshold effects for Ca, P, and Zn, where higher concentrations were associated with a lower likelihood of LMS, highlight the critical role of mineral homeostasis in supporting lactation. Ca and P are integral to milk synthesis as a key components of casein micelles [70], which ensures the stability and bioavailability of essential nutrients and influence physical properties of human milk. Zn is another vital mineral for lactation, serving as a cofactor for enzymes involved in cell differentiation, maintenance of a secretory phenotype, protein synthesis, and possibly secretion [71]. Zn levels in milk are regulated by Zn transporters, which actively transfer Zn into the mammary gland [72]. An animal study showed that the absence of Zn transporter ZnT2 reduced milk yield and decreased Zn, beta-casein, fat, and lactose concentrations in milk [21].

In this study, maternal BMI showed a positive association with the likelihood of LMS, with a sharp increase in LMS probability at BMI levels exceeding 25 kg/m^2^. This finding aligns with previous research linking maternal obesity to adverse lactation outcomes. Women with higher BMI are less likely to initiate, exclusively breastfeed, or continue breastfeeding [73]. A recent systematic review documented that women with overweight or obesity face both physical and psychological barriers to initiating and continuing breastfeeding, such as difficulties in positioning their infants for breastfeeding and body confidence issues [74]. Obesity is also linked to diverse endocrine dysregulations, including insulin resistance [75] and decreased prolactin activity [64,76]. Insulin metabolism plays a vital role in milk secretion, as it regulates genes involved in mammary epithelial cell proliferation and stimulates milk protein and lipid biosynthesis [75,77,78]. Given that prolactin is a major hormone promoting mammary gland differentiation and MP, reduced prolactin activity may lead to delayed and decreased lactogenesis and lower milk yield, as demonstrated in animal studies [79,80]. Further, we observed that women with LMS were significantly older than women with NMS, and maternal age was one of the important predictors in our machine learning models. Although controversial, increasing maternal age has been associated with a higher risk of non-exclusive breastfeeding, shorter breastfeeding duration and LMS [37,81,82,83], likely due to a reduced basal metabolic rate and decreased insulin sensitivity [84,85]. Lastly, infant WAZ was another significant predictive variable. Lower infant WAZ can be the consequence of inadequate milk intake, especially for infants not supplemented with formula despite their mothers having LMS. Among the mothers with LMS in this study, only 58.6% supplemented their infants with formula, which may explain why infant WAZ strongly predicted LMS in the model. This finding underscores the importance of monitoring infant growth trajectories, particularly if the mother and/or clinician suspects LMS.

The strengths of this study included the measurement of 24-h MP, comprehensive milk composition, and the extensive testing of prediction variables using various machine learning algorithms. Notably, the prediction model developed with milk components, anthropometrics, and demographics produced consistently favourable results and remarkably accurate predictions in a test set, suggesting that this could be a feasible approach to detecting LMS in practice. Given the development of portable point-of-care instruments, such as ion-selective electrode probes (ISEP) to measure minerals [46] and near-infrared spectrophotometry to measure macronutrient concentrations in human milk [45,47], using milk composition to predict LMS could become a time-efficient and cost-effective tool for clinical assessments. Lactation consultants working with women facing breastfeeding challenges could potentially use this tool to identify or confirm LMS promptly in the postpartum period. This approach offers a practical alternative to traditional methods of measuring MP, enabling more personalized care for breastfeeding mothers. A limitation of this study is the absence of comprehensive maternal metabolic health data covering the pre-pregnancy, pregnancy, and postnatal periods. Conditions such as pre-existing diabetes mellitus, GDM, PCOS, thyroid dysfunction, and anaemia are potential risk factors for LMS [86,87]. Incorporating this information would enhance the predictive models and better understand the relationships between milk composition and MP. While replicating performance in truly independent and larger samples will be necessary to rule out overfitting, our cross-validation and holdout testing approaches somewhat mitigate this concern in the available sample.

## 5. Conclusions

Our study has provided valuable insights into the associations between human milk composition and the likelihood of LMS. By developing machine learning models incorporating milk composition, demographic and anthropometric data, we achieved impressive accuracy in identifying women with LMS. These findings provide a robust evidence base for the identification of women at risk of LMS, facilitating timely interventions to support breastfeeding and ensuring infants receive adequate nutrition. Moving forward, research should focus on elucidating the biological mechanisms underlying LMS and testing the efficacy of predictive models in real-world settings.

## Figures and Tables

**Table 1:** Participant characteristics and milk composition.

Characteristics	NMS	LMS	*p*-Value
**Maternal and infant factors**			
MP (mL/24 h)	846 ± 208 (602–1682, 106)	416 ± 140 (81–597, 58)	**<0.001**
Infant age at MP measurement (months)	2.9 ± 1.1 (1.0–5.3, 106)	2.8 ± 1.2 (1.1–5.1, 58)	0.429
Infant age at sample collection (months)	3.1 ± 1.2 (1.0–5.4, 106)	2.9 ± 1.3 (1.0–5.3, 58)	0.466
Maternal age (years)	33.4 ± 4.5 (22.7–46.2, 106)	35.2 ± 4.4 (25.0–49.0, 58)	**0.014**
Maternal BMI (kg/m^2^)	28.2 ± 6.0 (19.5–64.5, 101)	28.9 ± 5.2 (19.4–42.9, 54)	0.223
Maternal BV growth during pregnancy (cm^3^)	191 ± 146 (0–730, 78)	170 ± 169 (0–700, 47)	0.161
Infant birth weight (g)	3446 ± 445 (2310–5040, 106)	3354 ± 475 (2270–5045, 58)	0.084
Infant WAZ	−0.3 ± 0.9 (−2.9–1.8, 100)	−0.7 ± 0.8 (−2.6–1.0, 54)	**0.004**
Infant sex: male	52 (49.1%)	28 (49.1%)	1.000
Parity: primiparous	65 (61.3%)	36 (62.1%)	1.000
Delivery mode: vaginal	58 (55.2%)	31 (54.4%)	1.000
Milk removal frequency (times/24 h)	13.4 ± 4.3 (4–28, 106)	12.4 ± 4.5 (5–24, 58)	0.115
Exclusive breastfeeding	96 (90.6%)	24 (41.4%)	**<0.001**
**Milk composition**			
Fat (g/L)	51.2 ± 17.6 (22.6–110.2, 106)	44.3 ± 15.8 (20.2–83.6, 58)	**0.020**
Lactose (g/L)	71.6 ± 24.3 (33.8–206.4, 106)	81.5 ± 15.8 (50.0–140.0, 58)	**<0.001**
Total protein (g/L)	13.1 ± 4.3 (3.1–26.6, 106)	11.7 ± 4.1 (4.4–23.6, 58)	0.055
Casein (g/L)	1.1 ± 0.5 (0.2–2.6, 106)	1.0 ± 0.6 (0.1–2.6, 58)	0.234
Whey protein (g/L)	8.6 ± 2.9 (2.6–22.9, 106)	8.0 ± 3.0 (1.1–20.1, 58)	0.160
Citrate (mg/L)	524.9 ± 271.7 (128.7–1227.5, 106)	363.6 ± 170.8 (121.0–781.8, 58)	**<0.001**
Ca (mg/L)	254.6 ± 60.7 (127.6–418.5, 106)	252.4 ± 50.3 (123.1–338.8, 58)	0.802
Cu (mg/L)	0.3 ± 0.3 (0.0–2.1, 106)	0.4 ± 0.3 (0.0–1.9, 58)	0.173
Fe (mg/L)	0.5 ± 0.4 (0.1–1.7, 106)	0.7 ± 0.9 (0.0–6.6, 58)	0.625
K (mg/L)	574.9 ± 131.0 (237.0–882.3, 106)	575.6 ± 127.5 (281.1–853.7, 58)	0.654
Mg (mg/L)	30.9 ± 6.0 (17.3–46.4, 106)	30.7 ± 6.4 (11.8–40.6, 58)	0.893
Na (mg/L)	126.4 ± 66.6 (23.1–413.6, 106)	143.9 ± 88.1 (29.7–663.2, 58)	0.063
P (mg/L)	139.1 ± 31.1 (68.2–225.7, 106)	132.8 ± 28.0 (55.5–207.3, 58)	0.192
Zn (mg/L)	1.9 ± 1.5 (0.3–6.3, 106)	2.1 ± 1.3 (0.3–5.1, 58)	0.238

BMI: body mass index; BV: breast volume; Ca: calcium; Cu: copper; Fe: iron; K: potassium; LMS: low milk supply; Mg: magnesium; MP: milk production; Na: sodium; NMS: normal milk supply; P: phosphorus; WAZ: weight-for-age z-score; bold font indicates statistically significant difference (*p* < 0.05); values are mean ± SD (range, *n*) or frequency (percentage).

## Data Availability

Restrictions apply to the availability of some or all data generated or analysed during this study. The corresponding author will, on request, detail the restrictions and any conditions under which access to some data may be provided.

## Supplementary Materials

The following supporting information can be downloaded at: https://www.mdpi.com/article/10.3390/diagnostics15020191/s1, Figure S1: multicollinearity evaluation. (A) Pairwise correlations; (B) variance inflation factors (VIF); Table S1: model performance metrics from cross-validation in the training set.
