# Supplementary material for: Milk Composition Is Predictive of Low Milk Supply Using Machine Learning Approaches
Source: Diagnostics (Basel). 2025 Jan 15;15(2):191. doi: 10.3390/diagnostics15020191 (PMC11764425; doi:10.3390/diagnostics15020191)
Supplement: Supplementary file 1 [file diagnostics-15-00191-s001.zip › diagnostics-3380939-supplementary.pdf]

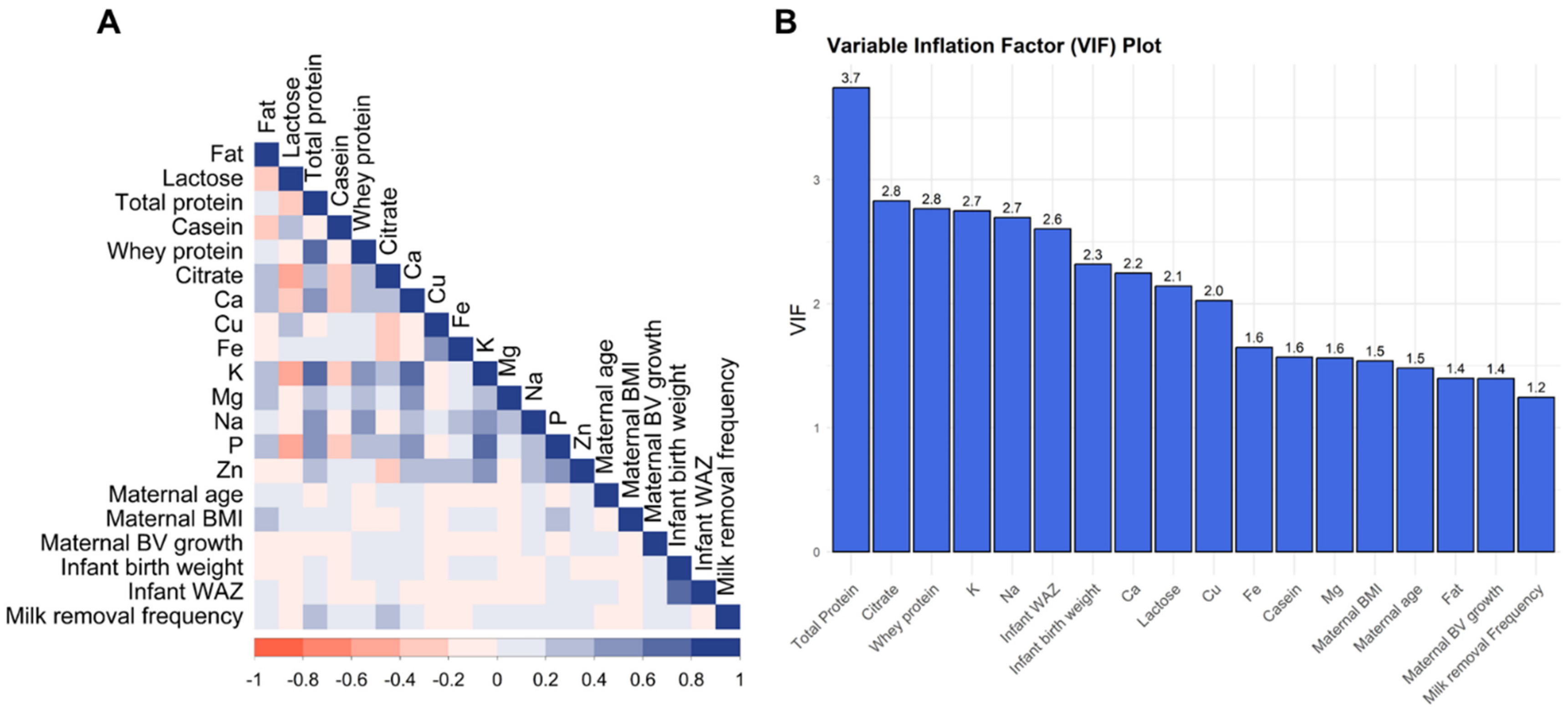

Figure S1. Multicollinearity evaluation. (**A**) Pairwise correlations. (**B**) variance inflation factors (VIF).

Table S1. Model performance metrics from cross-validation in the training set

Predictors	Algorithm	Accuracy	Precision	Recall	Specificity	F1	AUC	AUPRC
Predictor A	DL	0.892 ± 0.098	0.878 ± 0.118	0.946 ± 0.107	0.832 ± 0.187	0.902 ± 0.081	0.907 ± 0.128	0.913 ± 0.114
	XRT	0.854 ± 0.139	0.838 ± 0.148	0.944 ± 0.079	0.757 ± 0.294	0.878 ± 0.094	0.872 ± 0.138	0.899 ± 0.099
	DRF	0.848 ± 0.137	0.849 ± 0.154	0.922 ± 0.118	0.771 ± 0.297	0.869 ± 0.094	0.872 ± 0.145	0.886 ± 0.140
	GBM	0.825 ± 0.141	0.795 ± 0.150	0.956 ± 0.078	0.688 ± 0.294	0.858 ± 0.095	0.836 ± 0.138	0.840 ± 0.117
	XGBoost	0.763 ± 0.133	0.718 ± 0.123	0.968 ± 0.074	0.542 ± 0.291	0.816 ± 0.083	0.778 ± 0.190	0.810 ± 0.157
	GLM	0.722 ± 0.135	0.714 ± 0.160	0.912 ± 0.115	0.511 ± 0.363	0.782 ± 0.080	0.679 ± 0.212	0.711 ± 0.195
Predictor B	DL	0.915 ± 0.041	0.921 ± 0.076	0.924 ± 0.074	0.907 ± 0.093	0.918 ± 0.039	0.933 ± 0.036	0.924 ± 0.076
	XRT	0.859 ± 0.107	0.849 ± 0.129	0.911 ± 0.102	0.800 ± 0.190	0.872 ± 0.090	0.890 ± 0.114	0.903 ± 0.109
	DRF	0.854 ± 0.115	0.854 ± 0.128	0.900 ± 0.110	0.801 ± 0.224	0.867 ± 0.088	0.886 ± 0.098	0.905 ± 0.083
	GBM	0.859 ± 0.048	0.848 ± 0.072	0.900 ± 0.097	0.808 ± 0.124	0.868 ± 0.051	0.866 ± 0.081	0.876 ± 0.089
	XGBoost	0.847 ± 0.055	0.817 ± 0.091	0.933 ± 0.094	0.747 ± 0.150	0.864 ± 0.046	0.850 ± 0.076	0.853 ± 0.083
	GLM	0.739 ± 0.140	0.753 ± 0.177	0.878 ± 0.161	0.579 ± 0.378	0.783 ± 0.091	0.728 ± 0.165	0.743 ± 0.175
Predictor C	DL	0.695 ± 0.121	0.679 ± 0.135	0.903 ± 0.141	0.475 ± 0.312	0.757 ± 0.080	0.661 ± 0.125	0.673 ± 0.143
	XRT	0.842 ± 0.107	0.829 ± 0.126	0.911 ± 0.070	0.765 ± 0.217	0.861 ± 0.076	0.867 ± 0.107	0.880 ± 0.109
	DRF	0.853 ± 0.095	0.830 ± 0.113	0.934 ± 0.077	0.767 ± 0.208	0.872 ± 0.068	0.887 ± 0.092	0.899 ± 0.094
	GBM	0.825 ± 0.127	0.796 ± 0.144	0.947 ± 0.056	0.699 ± 0.270	0.856 ± 0.087	0.851 ± 0.114	0.858 ± 0.108
	XGBoost	0.864 ± 0.055	0.834 ± 0.071	0.934 ± 0.093	0.788 ± 0.120	0.877 ± 0.050	0.865 ± 0.078	0.871 ± 0.076
	GLM	0.785 ± 0.095	0.765 ± 0.109	0.889 ± 0.128	0.668 ± 0.232	0.812 ± 0.076	0.765 ± 0.121	0.769 ± 0.137

AUC: area under the receiver operating characteristic curve; AUPRC: area under the precision-recall curve; DL: deep learning; DRF: distributed random forest; GBM: gradient boosting machine; GLM: generalized linear model; XGBoost: extreme gradient boosting; XRT: extremely randomized trees.
